# Efficacy of either orally administered fluralaner or topically administered imidacloprid/flumethrin for controlling *Rhipicephalus sanguineus* sensu lato premises infestations

**DOI:** 10.1186/s13071-023-06028-0

**Published:** 2023-11-14

**Authors:** Marcelo Bahia Labruna, Juliana Salomão Doretto, Olivia Carmen de Araújo Nascimento, Francisco Bonomi Barufi, Simone Cristina Rosa, Germana Vizzotto Osowski, Jason Drake, Rob Armstrong

**Affiliations:** 1 Parasitic Disease Laboratory, School of Veterinary Medicine and Animal Science, University of São Paulo, São Paulo, Brazil; 2 Centro de Pesquisa de Animais do Brasil, Santo Antônio de Posse, Brazil; 3 MSD Animal Health, São Paulo, Brazil; 4 Merck Animal Health, Rahway, NJ 07940 USA; 5 Laboratory of Epidemiology and Biostatistics, School of Veterinary Medicine and Animal Science, University of São Paulo, São Paulo, Brazil

**Keywords:** Acaricide, Dog, Rhipicephalus, Fluralaner, Flumethrin

## Abstract

**Background:**

Adult, nymph, and larval *Rhipicephalus sanguineus* sensu lato infest dogs and thrive in premises including homes and kennels. Ticks emerge from hiding to seek and attach to dogs, engorge, then leave their hosts to hide then molt or oviposit. This study evaluated the effect of either external or systemic canine treatment on *R. sanguineus* s.l. populations in premises.

**Methods:**

Thirty-two dogs in eight kennels were divided into three groups; one group (eight dogs in two kennels) served as untreated controls; one group (12 dogs in three kennels) received oral fluralaner (25–56 mg/kg); and one group (12 dogs in three kennels) received topical flumethrin/imidacloprid impregnated collars. Treatments were administered once on day 0. Prior to treatment, *R*. *sanguineus* s.l. infestations were established in kennels holding dogs, by placing ticks every 2 weeks from day −84 through day −14. Kennel surfaces (walls and floors) were smooth except for uniform “hideouts” to permit precise off-host tick counting.

**Results:**

Control dog kennel mean tick counts (all life stages) increased from 737 ticks/kennel at day −7 to 2213 at day 63 when all control kennel dogs were acaricide-treated as a humane endpoint. Kennels housing dogs subsequently treated with systemic fluralaner had a mean of 637 counted ticks/kennel on study day −7 (7 days before treatment). One fluralaner treatment eliminated all premises ticks (100% reduction) by day 70, and these kennels remained tick-free through study completion (day 84). Kennels housing dogs subsequently treated with an external imidacloprid/flumethrin collar had a mean of 614 counted ticks/kennel at study day −7. Collar treatment reduced counts by 90% on day 63, with kennel tick counts climbing after this and ending the study with a 75% reduction. Systemic fluralaner treatment was significantly (*P* = 0.003) more effective at reducing engorged adult female tick counts than external imidacloprid/flumethrin treatment.

**Conclusions:**

Fluralaner treatment eliminated off-host *R. sanguineus* life stages in infested kennels by day 70 following treatment and was significantly more effective than imidacloprid/flumethrin collar treatment in reducing the premises population of engorged female ticks. Imidacloprid/flumethrin treatment did not eliminate premises tick populations, with populations increasing before the study end.

**Graphical Abstract:**

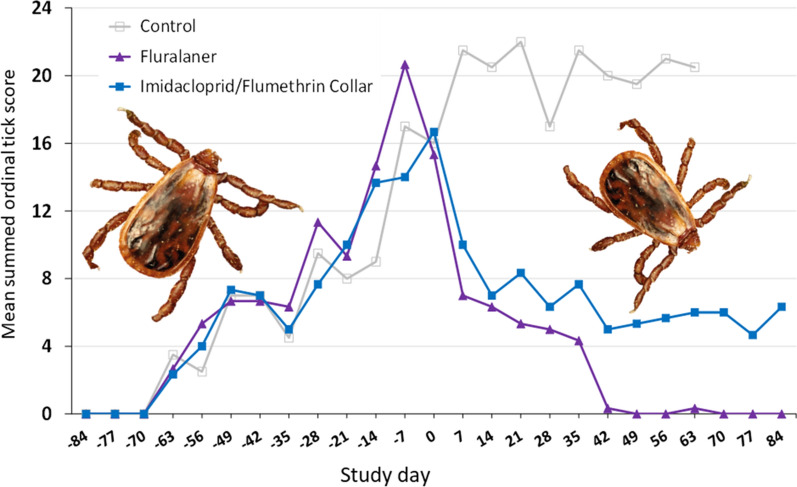

## Background

*Rhipicephalus sanguineus* sensu lato (s.l.), commonly called the brown dog tick or the kennel tick, is a serious problem for dogs and their owners. This tick colonizes both human and canine dwellings and reproduces rapidly when conditions are favorable [1]. *Rhipicephalus sanguineus* has a three-host life cycle, in which the three motile life stages (larva, nymph, adult) feed preferentially on dogs and ingest blood. The resulting blood loss can cause severe and potentially fatal anemia [2]. In addition, life stages feeding on domestic dogs can transmit infectious organisms, particularly the bacterium *Ehrlichia canis,* the etiological agent of canine monocytic ehrlichiosis, and the protozoan *Babesia vogeli,* the etiological agent of canine babesiosis [2]. This tick also occasionally feeds on humans, and is implicated in the transmission of the bacterium *Rickettsia rickettsii,* the etiological agent of Rocky Mountain spotted fever [3, 4].

In homes, apartments, and kennels, *R. sanguineus* is usually present around the bedding area of dogs [1]. After feeding for a few days (3–6 days for larvae and nymphs, 6–9 days for adult females) on dogs, engorged ticks detach themselves, preferably at night [5], and migrate toward cracks or crevices, or between walls, where engorged larvae and nymphs molt to nymphs and adults, respectively, and engorged females each oviposit several thousand eggs in a cluster [1]. This nidicolous behavior and the habits of engorged ticks to search for hidden places reduces *R. sanguineus* vulnerability to environmental humidity fluctuations and explains its ability to become established within households in both high humidity areas such as the Amazon [6], or desert areas such as northern Mexico [7].

The *R. sanguineus* life cycle is highly temperature dependent, and off-host developmental time is dramatically shortened by warmer temperatures [8] as demonstrated in a southeastern Brazil (Minas Gerais state) *R. sanguineus* population under laboratory conditions. Engorged larvae molted to nymphs (pre-molt period) in a mean of 7.5 weeks at 16 °C and < 2.0 weeks at 24 °C; engorged nymphs molted to adults in 25.5 weeks at 16 °C and 3.5 weeks at 24 °C; engorged females started oviposition (pre-oviposition period) in 6 weeks at 16 °C and 1 week at 24 °C, and larvae hatched from eggs (incubation period) after 4.2 weeks at 24 °C with no larval hatching at 16 °C [8]. This temperature effect leads to higher loads of *R. sanguineus* ticks on dogs during spring and summer months, although dogs can be infested year-round among tropical and subtropical regions [9–11].

The tremendous household adaptation of *R. sanguineus* makes elimination very difficult, especially in urban areas, because off-host life stages infest hidden premises locations [12]. Failure to eliminate all the hidden premises tick stages during acaricidal treatment results in the rapid rebuild of the tick infestation once treatment is discontinued. Therefore, effective acaricides that also eliminate premises life stages must be used to successfully treat dogs for these ticks. Effective acaricide options for dogs include topical and systemic treatments [13]. The objective of this study was to evaluate the efficacy of two different treatments for controlling life stages of *R. sanguineus* ticks in the kennel premises. One treatment is a long-acting systemic treatment—fluralaner (Bravecto^®^, Merck Animal Health, Rahway, NJ, USA), and the other is a topical repellent treatment—imidacloprid/flumethrin combination delivered via a long-lasting collar (Seresto^®^, Elanco Animal Health, Indianapolis, IN, USA). The study site simulated a natural kennel infestation by introducing ticks and providing hiding places for tick reproduction. Untreated control kennels provided a comparative evaluation of untreated tick population growth under these conditions.

## Methods

### Study site

This study was performed in an animal experimental facility located in a rural area of São Paulo, southeastern Brazil. The study area has a tropical savanna climate characterized by warm/hot and rainy summers (average temperature 24 °C), with mild and dry winters (average temperature 18 °C). The present study was conducted from late winter (September 2022) to late summer (February 2023).

### Experimental kennels

**Fig. 1:**
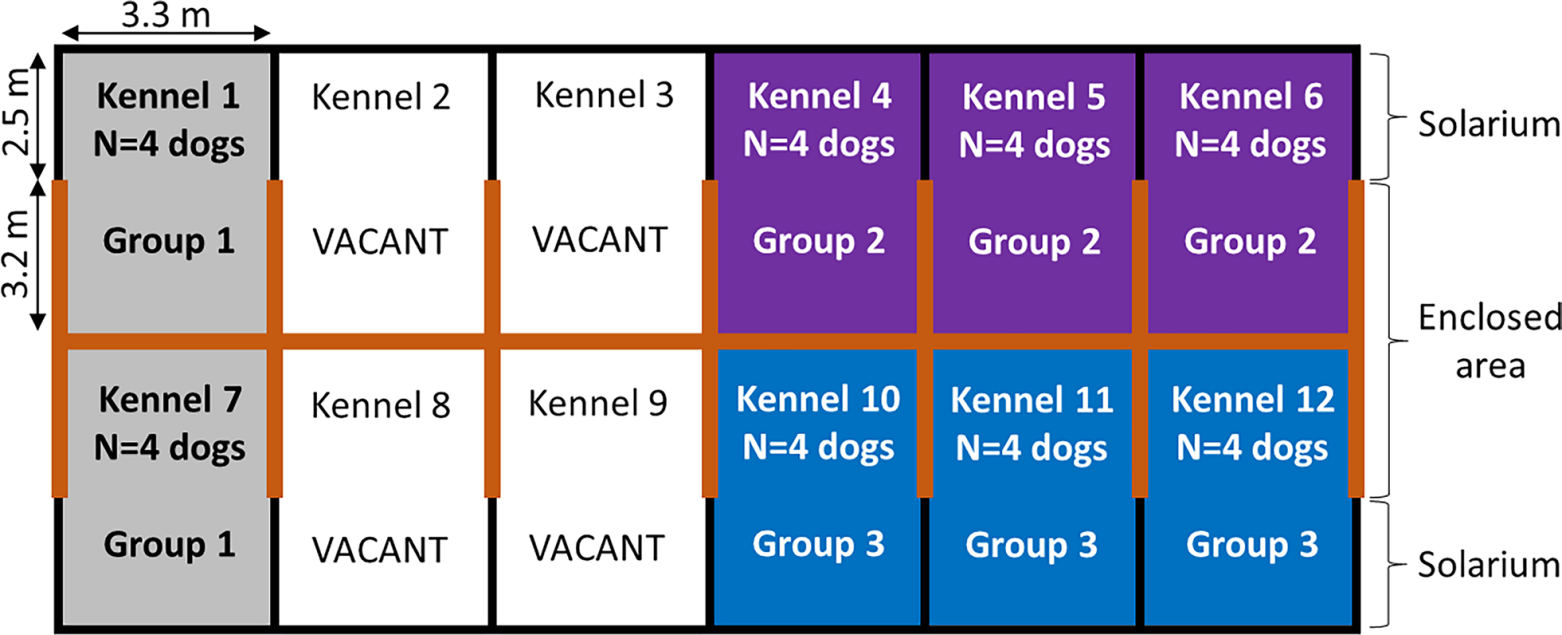
A schematic diagram of kennel layout and dimensions for a study evaluating acaricide efficacy for control of *Rhipicephalus sanguineus* s.l. premises life stages

The eight kennels in the study were part of a 12-kennel unit kept under ambient conditions subject to seasonal variation. Each kennel consisted of a 10.56 m^2^ enclosed area (3.3 m × 3.2 m) and an 8.25 m^2^ open area, i.e., solarium (3.3 × 2.5 m), both with a concrete floor and connected by a 1.2-m-wide open door on one side (Fig. 1). The top of each kennel was the same height in all kennels at the front (2.7 m) and the back (3.4 m). The enclosed portion of the kennel provided sun protection and a natural photoperiod was maintained without artificial lighting. There were no interior communicating openings between kennels and walls. In the open-air area, kennels were separated by a small wall containing a wire fence; the external walls were smeared with a petroleum jelly tick barrier. Kennels contained several wood beds which, together with the concrete floor, were washed every day to remove canine waste. There were two kennels assigned to control dogs and six kennels assigned to hold treatment group dogs, with three kennels per treatment group. Kennel assignment within the unit maintained unoccupied kennels (vacant) between control and treatment groups to prevent potential tick movement from control to treatment kennels with both treatment groups equidistant from control group kennels.

Identical tick hideouts used to quantitate premises tick infestations were created and marked out in all enclosed kennel areas. In addition, kennels were carefully inspected to ensure that no crack or hole remained to serve as an alternative tick hideout. Hideouts were individually numbered to identify the kennel location, and all were identically located and each occupied 900 cm^2^. All tick life stages in the hideouts were counted.

### Dogs

The study included 32 adult beagles, 1–8 years old, either male or female, weighing between 6.0 and 20 kg. Dogs were dewormed and assessed as healthy before the study start and were not tick- or flea-infested. Dogs were acclimatized in the facility for 7 days before starting the study at day −84 (negative 84, indicating 84 days before treatment administration on day 0). Five days before starting the study (day −89), dogs were ranked on *R. sanguineus* s.l. carrying capacity by challenging each dog with 20 unfed adult tick pairs (from the same tick colony mentioned below), which were manually removed from the dogs and counted 4 days later (day −85) and based on the results dogs were assigned to four blocks of eight dogs. One dog from each block was randomly assigned to each of the eight kennels resulting in four dogs per kennel. An additional six dogs meeting study inclusion criteria were held in a separate facility as potential substitutions into study kennels if needed.

### Artificial infestations of kennels

There were no ticks in any site kennel before the study, and the unit had not housed dogs for more than 5 years. Engorged ticks were laboratory-bred for release into the kennels during the study to establish the premises infestation. These ticks were derived from a *R. sanguineus* s.l. laboratory colony recently established from engorged females collected from naturally infested dogs in Minas Gerais state (altitude 850 m), Brazil, ≈400 km from the study site. Tick colony progenitors were demonstrated to be free of DNA of *Anaplasmataceae* (*Ehrlichia* spp., *Anaplasma* spp.), *Babesia* spp., and *Rickettsia* spp. through specific polymerase chain reaction (PCR) test protocols [14].

Engorged ticks were obtained through artificial infestations on naïve rabbits in the laboratory and transported to the kennel site for release into kennels within 24 h after detachment from the initial rabbit hosts. A total of six engorged tick releases were performed in the kennels, every 2 weeks, on days −84 (study start), −70, −56, −42, −28, and −14. No additional ticks were released following treatment administration on day 0. Ticks released in each kennel at each of the six time points comprised equal numbers per kennel of engorged larvae, engorged nymphs, and engorged female adults. Released ticks were free to search the kennel for suitable molting sites (engorged larvae and nymphs) or ovipositing sites (engorged females) in tick hideouts. This procedure created an established *R. sanguineus* s.l. population in each kennel, where recently molted ticks (unfed nymphs and adults) and hatched larvae could actively feed on the housed dogs, and then return to the hideouts.

At each of the six time points of tick release in the kennels, an ecdysis/oviposition monitoring test was run to document successful molting, oviposition, or hatchability of engorged ticks placed in kennels. Successful monitoring test results proved that kennel conditions supported tick molting and oviposition and indicated when challenge ticks would begin infesting dogs. For this test, 20 engorged larvae, 10 engorged nymphs, and three engorged tick females (all from the same cohort of ticks released at that time point) were placed in a separate labeled plastic tube (1.2 × 8 cm with perforated cap). Tubes were placed in one kennel (a different kennel for each release date) to maintain similar environmental conditions to those experienced by released challenge ticks. Placed tubes were inspected every 3 or 4 days to record the time of either ecdysis from one tick life stage to the next (larvae to nymphs, nymphs to adults), oviposition, or larval hatching. Molting or oviposition success was calculated for each tube giving the percent of engorged ticks that successfully completed development to the next stage. Percent (%) hatching was visually estimated for each oviposited egg mass. A pre-hatching period was calculated as the number of days between the placement of a tube containing engorged females and first appearance of hatched larvae in the tube.

### Experimental groups

The study objective was to quantitate premises (off-host) tick populations and not dog infestations; however, dogs were essential in the study to provide tick hosts and simulate a natural tick infestation. Dogs were held continuously in kennels throughout the study, from day −84 (late winter) until day 84 (midsummer). Kennels 1 and 7 held untreated control dogs; dogs in kennels 4, 5, and 6 were treated on day 0 with oral fluralaner according to product label directions for the weight of the dog; and dogs in kennels 10, 11, and 12 were treated on day 0 with topical imidacloprid/flumethrin as a collar administered according to product label directions for the weight of the dog. If a collar was lost or removed from a topically-treated dog, then a new collar was placed on that dog as soon as the loss was observed. All products were purchased from the same source and no other substances with potential insecticidal or acaricidal activity, such as medicated shampoos, were used on the dogs or the kennels during the study. All dogs had a hematocrit measurement every 2 weeks to look for initial signs of potential blood loss from tick feeding. Any dog experiencing clinical signs of ill health caused by tick infestation or any other cause was removed from the study, treated, and replaced by one of the additional six dogs held in a separate facility as potential substitutions.

### Tick counts

The inclusion of a visible collar treatment in one treatment group precluded masking for investigators counting premises ticks. Premises ticks were counted in all eight kennels every 7 days throughout the study period, beginning at the time of initial challenge on study day −84. For the count, tick hideouts were closely examined and all visible tick life stages were counted. There were 16 tick hideouts in each kennel and eight of these were counted at each counting period. Hideouts counted were alternated so that no location was disturbed 2 weeks in a row and remaining hideouts were undisturbed between counts. Counted ticks were not touched or removed from the kennels or hideouts.

Counted ticks were classified by tick life stage and developmental stage, as follows: engorged females (not started oviposition); ovipositing females (ovipositing with no larval hatch); unfed larvae (counted as a cluster of unfed larvae, usually observed next to egg remnants from a former ovipositing female); engorged larvae; unfed nymphs; engorged nymphs; and unfed adults (no discrimination between males and females). Counted ticks were not classified as live or dead because they were expected to be immobile; however, ticks showing unambiguous signs of death (e.g., blackish engorged females; withered and dull ticks) and non-viable tick detritus (shed exoskeletons, eggshells) were not counted. Tick stages were counted individually except for unfed larvae counted as larval clusters.

Feeding ticks on dogs were not disturbed or removed and tick presence was noted using an ordinal classification. The entire body of each dog was examined every 7 days, and attached ticks were classified into life stage (larvae, nymphs, adults), with each stage enumerated as follows: 0 (no ticks), 1 (1–20 attached ticks), or 2 (> 20 attached ticks). The maximum possible ordinal tick presence on dogs score for a kennel in a counting date was 24, comprising > 20 attached larvae (score = 2), > 20 attached nymphs (score = 2) and > 20 attached adults (score = 2) on all four dogs [(2 + 2 + 2) × 4 dogs = 24)].

### Climatic data

Daily temperature and rainfall data for the study period were downloaded from the public website of the Instituto Nacional de Meteorologia [15]. The nearest available station is 22 km from the study site. Temperature data are presented as the mean of the previous 7 days, and rainfall data are presented as the sum of the previous 7 days for 1 week before the study plus the 24-week study period.

### Statistical analyses

The compare groups of growth curves (CGGC) method was used, which performs permutation tests to assess differences in growth curves between groups using a kennel as the experimental unit. Calculations were performed by the compareGrowthCurves function (R Statistical Software v4.2.3; R Core Team 2021) [16, 17]. Growth curves were considered significantly different if *P* < 0.05. Mean counted ticks in hideouts of all experimental groups (control and two treatments) were compared over two study phases: from day −84 to day 0 (before treatment), and from day 0 to day 63 (the control group was discontinued on day 63—see Results). A third growth curve comparison was performed for mean tick counts of all life stages in treated dog kennels from day 0 to day 84, and this comparison analysis was also performed separately for each developmental stage (engorged females, ovipositing females, unfed larvae, engorged larvae, unfed nymphs, engorged nymphs, and unfed adults).

## Results

**Fig. 2:**
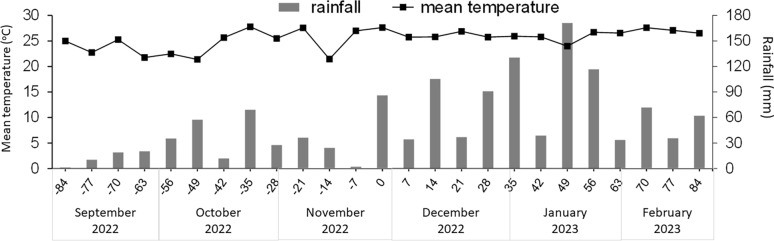
Outdoor temperature and rainfall during the study period of an evaluation of premises treatment of *Rhipicephalus sanguineus* s.l. infesting a kennel in Brazil

Study climatic data (Fig. 2) show that mean temperature remained between 21 and 27 °C during the pre-treatment period (days −84 to 0), then increased to between 24 and 27 °C) during the post-treatment period (days 0 to 84). Rainfall occurred throughout the study, although at higher volumes during the post-treatment period.

All treatments administered on day 0 were well tolerated by all study dogs with no reported adverse effects. However, the imidacloprid/flumethrin collar was removed by one dog at day 38; and was replaced with a new collar the same day.

### Premises tick counts

**Fig. 3:**
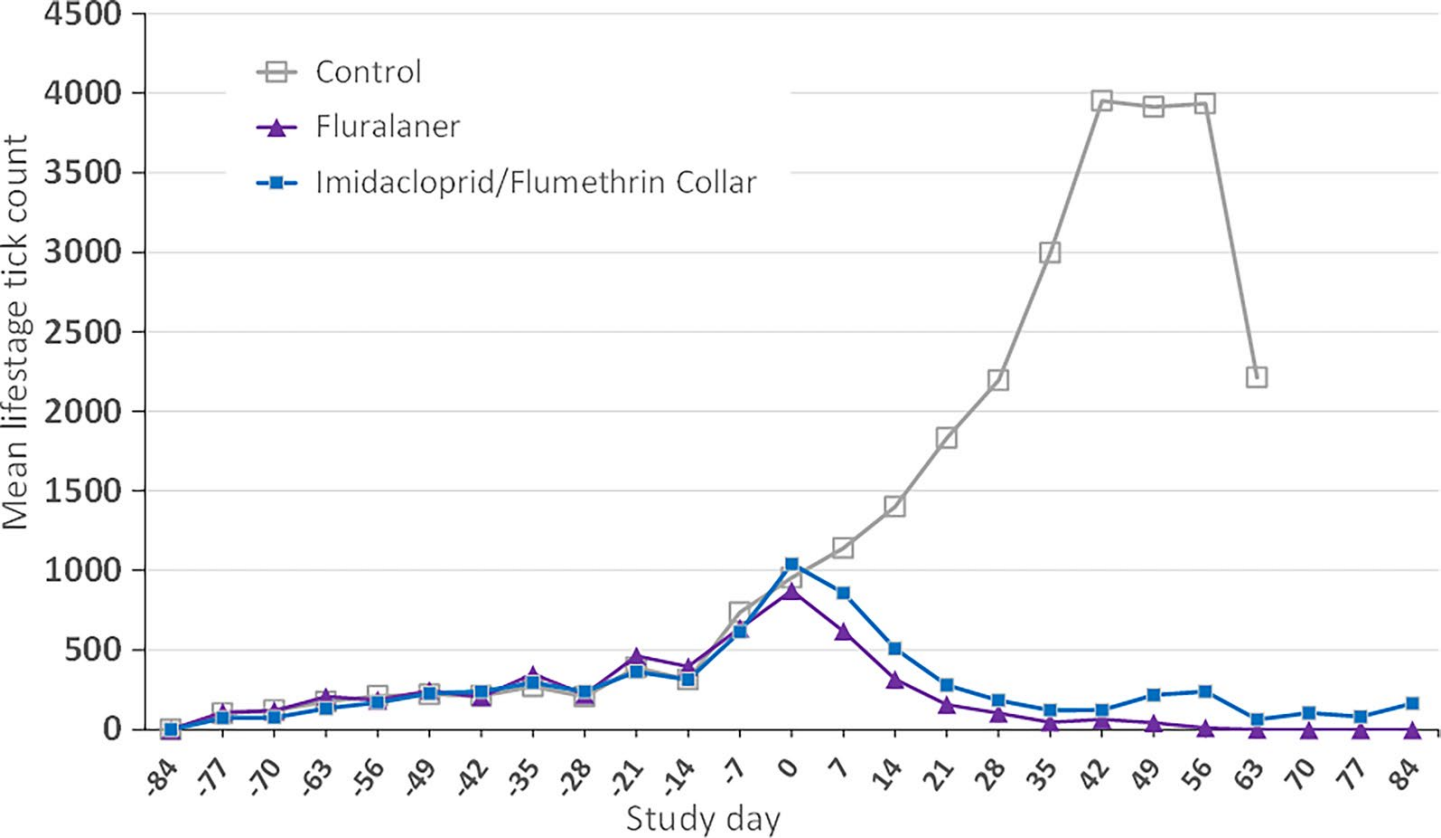
Mean premises total *Rhipicephalus sanguineus* s.l. life stage counts in kennels holding untreated control, fluralaner-treated or imidacloprid/flumethrin collar-treated dogs

Total premises tick count growth curves were not significantly different between groups during the pre-treatment study period of day −84 through treatment on day 0 (Fig. 3) and the initial count on day −84 was zero for all study kennels. Mean tick counts increased consistently in all groups from day −77 (mean tick counts varying from 73 to 109 ticks/kennel) until day 0 (mean tick counts from 873 to 1042 ticks/kennel). During this period there were six tick releases at 14-day intervals, and an approximately tenfold increase in the number of counted premises ticks in the kennels.

After treatment administration on day 0, premises tick counts for treated groups diverged from the control group, with the control group total premises tick counts continuing to climb even more steeply. Control dog kennel mean tick counts increased from 736 ticks/kennel at day −7 to close to 4000 ticks on day 42 and then plateaued before declining to 2213 ticks/kennel at day 63. The plateau at day 42 was associated with the humane removal of one heavily infested dog. Subsequently at day 63, the study was terminated for all control kennel dogs and they were removed and acaricide-treated as a humane endpoint because remaining control dogs had decreasing hematocrit.

Over the same period, premises tick counts in both treated groups declined with the lowest number of ticks always observed in the fluralaner-treated dog group. Kennels holding dogs to receive fluralaner treatment had a mean of 873 counted ticks/kennel on the day of treatment administration (day 0), then a single oral fluralaner treatment resulted in steady premises tick population reduction, with one tick seen in one kennel on day 63 and complete (100%) premises tick elimination by day 70. Kennels holding fluralaner-treated dogs remained tick-free through study completion (day 84).

Kennels holding dogs to receive imidacloprid/flumethrin collars had a mean of 1041 premises ticks/kennel on the day of treatment administration (day 0). Treatment of these dogs with a flumethrin/imidacloprid collar reduced premises counts by 90% on day 63, although mean premises tick counts then increased, and these kennels ended the study with a 75% reduction. Imidacloprid/flumethrin treatment never completed eliminated premises ticks at any point in the study. Recently engorged ticks are seen in the hideouts on the internal walls of kennels holding imidacloprid/flumethrin-treated dogs at day 70.

**Fig. 4:**
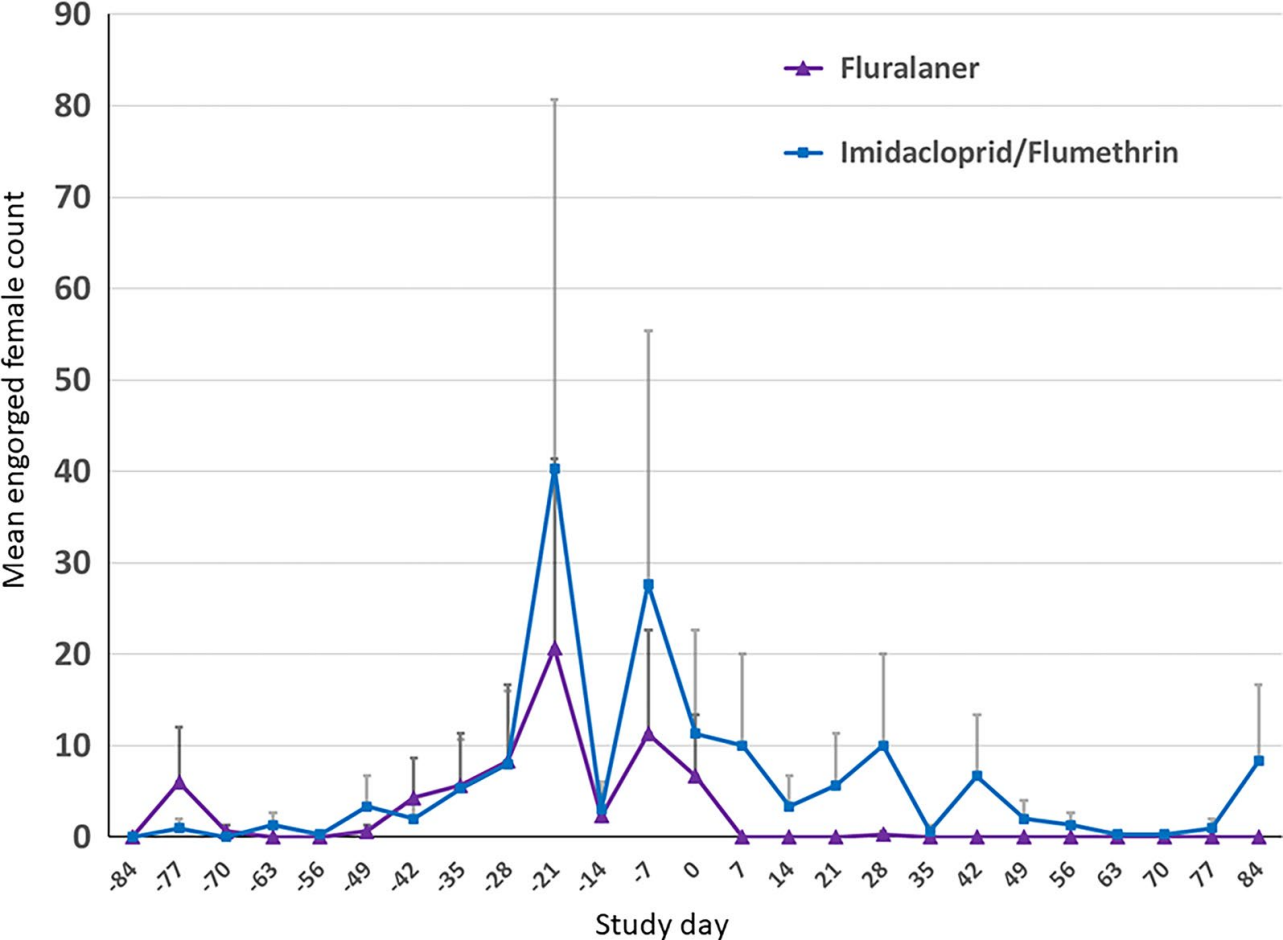
Mean premises *Rhipicephalus sanguineus* s.l. engorged female counts in kennels holding fluralaner-treated or imidacloprid/flumethrin collar-treated dogs. Vertical gray bars represent the standard deviation

Statistical analyses found that tick premises population growth curves in all kennels were not significantly different (*P* > 0.6) from day −84 to day 0. From day 0 to 63, the premises tick population growth curve of kennels holding untreated control dogs ticks was significantly different (*P* < 0.0001) from the kennels holding treated dogs. The premises tick population curves of the treated groups were not significantly different (*P* = 0.3) although there was a decrease of 1.18 in fluralaner-treated dog premises counts compared with imidacloprid/flumethrin counts. Tick developmental stages were compared separately between fluralaner and imidacloprid/flumethrin groups and there was a significantly lower growth curve for engorged female ticks compared with kennels holding imidacloprid/flumethrin-treated dogs (*P* = 0.003) (Fig. 4).

### Ecdysis/oviposition monitoring

Engorged larvae, nymphs, and females in the ecdysis monitoring tests were viable at all time points, with 100% molting, oviposition success, and egg mass hatch (Table 1). Pre-molt periods took longer for earlier tick release time points (14–24 days for larval pre-molt period; 21–31 days for nymphal pre-molt periods) than for later tick release time points (7–10 days for larval pre-molt period; 14–21 days for nymphal pre-molt periods), coinciding with an overall lower mean temperature in the early period and a higher mean temperature later (Fig. 1). A similar trend was observed for the pre-hatch period, with the shortest (17 days) observed on the last tick release time point on day −14. Unfed nymphs survived within the tubes after molt for 31 to 91 days, unfed adults for 38 to over 100 days, and unfed larvae for 77 to 87 days. For releases on days −42 and −28, some unfed adults remained alive in the tubes at the end of the study on day 84; therefore, their maximum survival was recorded as > 126 or > 112 days.

**Table 1:** Outcomes for engorged ticks (larvae, nymphs, or adult females) placed within ecdysis monitoring tubes in a different kennel at each tick release day and observed every 3–4 days for molting, oviposition, larval hatching or unfed tick survival

Day	Engorged larvae			Engorged nymphs			Engorged females			
	Molt success (%)	Pre-molt period (days)	Unfed nymph survival (days)^a^	Molt success (%)	Pre-molt period (days)	Unfed adult survival (days)^a^	Oviposition success (%)	Egg mass hatch (%)	Pre-hatch period (days)^b^	Unfed larva maximum survival (days)
−84	100	14−24	84–91	100	28–31	87–98	100	100	42–49	87
−77	100	14−17	73–77	100	24–31	77–101	100	100	32–38	87
−56	100	14	70–77	100	21–31	70–133	100	100	31	84
−42	100	14−17	56–77	100	17	56 to > 126^c^	100	100	31	77
−28	100	7−10	42−63	100	17−21	52 to > 112^c^	100	100	31	94
−14	100	7−10	31−49	100	14	38–80	100	100	17	77

^a^Death of first and last specimen within each tube

^b^Pre-hatch period—the days between tube placement containing engorged females and the first hatched larvae appearance

^c^Few unfed adults were alive on day 84; therefore, the maximum survival of these unfed adults was higher than the maximum value here

### Ticks on dogs

**Fig. 5:**
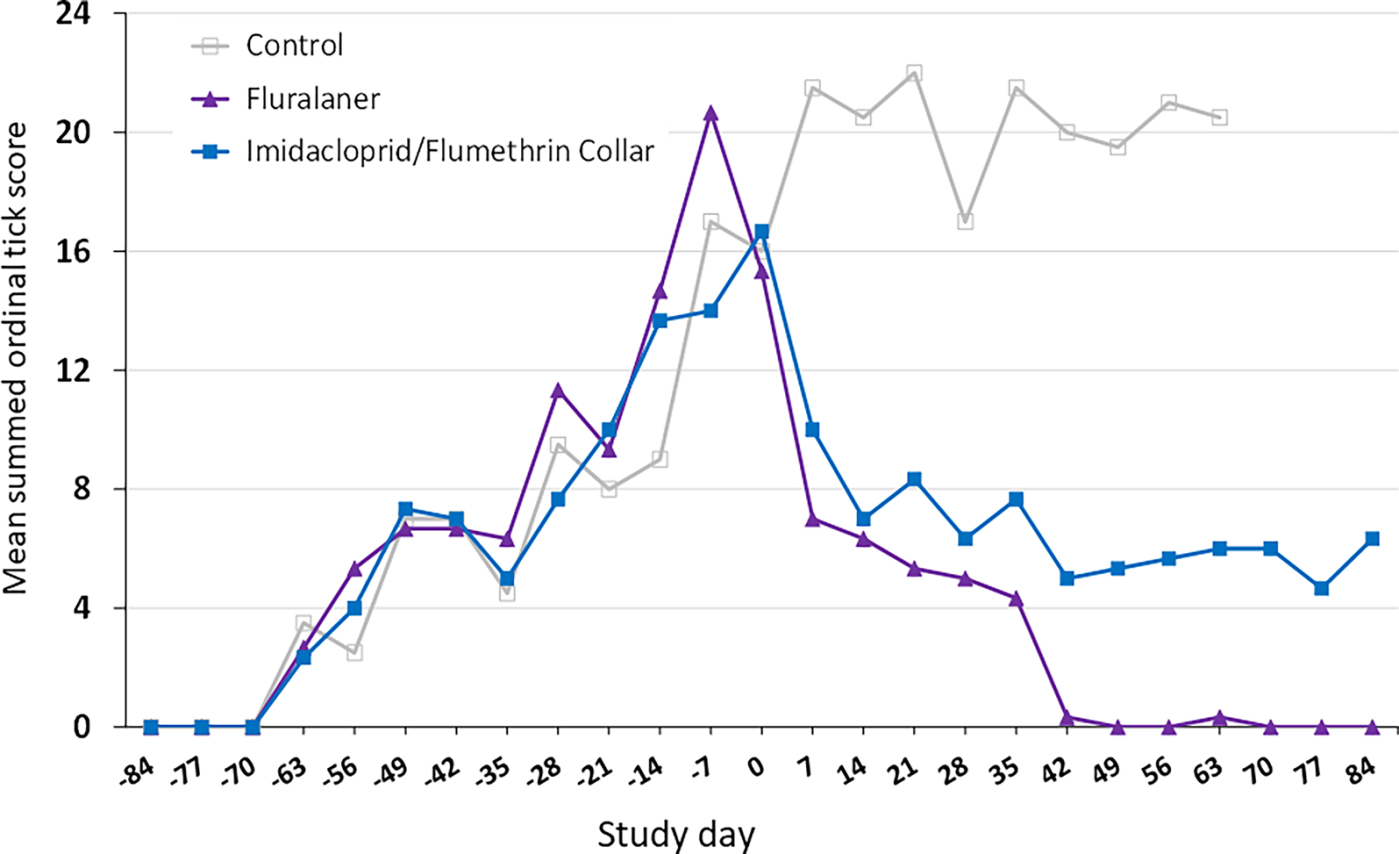
Mean summed ordinal *Rhipicephalus sanguineus* s.l. scores for dogs in a simulated kennel infestation and either untreated or treated with fluralaner or an imidacloprid/flumethrin collar on day 0

Summed ordinal scores for attached larvae, nymphs, and adult ticks on dogs from untreated controls and fluralaner or imidacloprid/flumethrin-treated dogs (Fig. 5) show that the first visible ticks attached on dogs appeared on day −63 in all kennels, and these were nymphs infesting dogs 21 days after the first tick release into kennels. This harmonizes with the 14-day-minimum pre-molt period of engorged larvae in monitoring tubes from day −84 (Table 1). The first attached adults on dogs were seen on day −56, 28 days after the first tick release and this is consistent with the 28-day-minimum pre-molt period of engorged nymphs in the monitoring tubes from day −84 (Table 1). Attached larvae were first seen on dogs on day −28, 56 days after the first tick release into kennels, also consistent with the 42−49-day pre-hatching period in the monitoring tubes placed day −84 (Table 1).

Mean summed ordinal tick score values for untreated and treated groups increased in an identical pattern from day −63 to treatment administration on day 0 (Fig. 5). Untreated control dog mean scores continued to increase after day 0, reaching a plateau around 20 until day 63 when all dogs of this group were withdrawn from the study and acaricide-treated as a humane endpoint. Fluralaner-treated dogs had a mean ordinal score of 21 pre-treatment on day −7, then after treatment on day 0 mean ordinal scores for this group dropped rapidly to close to 0 from days 42 to 84. Imidacloprid/flumethrin collar-treated dogs had a mean ordinal score of 14 at day −7, then after treatment with imidacloprid/flumethrin collars on day 0 the mean ordinal score dropped to values between 4 and 8 from days 14 to 84 although never lower than the score for the fluralaner-treated dogs and showing a slight increasing trend over the final weeks of the study.

## Discussion

This study demonstrated that effective acaricide treatment of actively *R. sanguineus*-infested dogs significantly reduced the off-host premises tick population throughout the treatment period. This treatment period was during the spring and summer months when the conditions were most favorable to rapid tick reproduction and infestation. Furthermore, systemic fluralaner treatment led to the elimination of off-host premises ticks, although topical imidacloprid/flumethrin collar treatment did not eliminate premises ticks, and after initial declines, premises tick populations in this group showed evidence of increase by the end of the study period.

*Rhipicephalus sanguineus* s.l. release at regular intervals into kennels holding dogs resulted in a dramatic growth in the off-host premises tick population. Total tick populations in kennels holding untreated control dogs increased continuously from day −14 through humane removal and replacement of one heavily infested dog and replacement on day 42. High tick viability and rapid tick reproduction compromise the health of untreated dogs based solely on blood loss without considering any additional impact of tick-transmitted disease as would be likely with a natural tick infestation.

The infestation level of ticks on the dogs (Fig. 5) presents a very similar growth pattern to the changing premises population of ticks in the kennels (Fig. 2). In addition, ticks counted on dogs are attached, and this means that ticks in the imidacloprid/flumethrin-treated group are engorging despite administering treatment with a reported repellent effect. These results also show that the tick population growth in the premises is rapidly reflected rapidly by changes in dog tick infestation levels and that ticks do not remain hidden in the premises despite either treatment. Estimating the tick infestation level in the dog could be an approach for estimating the premises tick infestation level—this is a point worth further investigation.

A weakness of this study design is the low statistical power because the experimental unit is the individual kennel and there were only three kennels per treatment group and two untreated control kennels. However, even considering the limited power of the study, both treatment groups showed a significant decline in total premises tick populations compared with untreated controls. In addition, the engorged female populations in the premises declined significantly in kennels holding fluralaner-treated dogs compared with imidacloprid/flumethrin-treated dogs. This difference in impact on engorged female ticks could be the result of lower susceptibility of adult ticks to topical acaricides [18, 19]. An alternative hypothesis could be that engorged female ticks are able to find feeding locations unprotected by a topically administered treatment, while there are no such locations on a dog following administration of a systemically distributed treatment [13]. The engorged female is in the multiplying stage of the tick life cycle because she oviposits thousands of individuals creating the next generation; therefore, this observed treatment success difference is likely an important factor in ongoing premises tick population control. This difference could explain the increase in tick premises populations observed in the later weeks of the study in kennels holding imidacloprid/flumethrin collar-treated dogs.

These results indicate that the treatment of choice for controlling *R. sanguineus* s.l. ticks in a home or kennel would be fluralaner administration because only fluralaner administration led to the complete elimination of premises tick populations and provided sufficiently extended tick adulticide effect following a single administration. Tick infestation recurrence following this treatment would depend on subsequent re-infestation of the kennels with a new tick population. Imidacloprid/flumethrin collar administration reduced tick populations but never eliminated them. Therefore, tick populations remained and could rapidly increase in these kennels based on the growth in the population of engorged females, although the study duration did not extend to the 8-month recommended retreatment interval for the imidacloprid/flumethrin collar, and further seasonal effects in either group were not evaluated. This study also did not evaluate any potential for resistance to flumethrin in the tick population. The study outcome showing a superior effect for a systemic acaricide is consistent with the prediction hypothesized in an earlier review of topical compared with systemic ectoparasite treatment [13]. Ticks infesting kennels holding topical repellent-treated dogs did not stay in the kennels hunting for an unprotected dog to feed on—these kennels also had a reduction in tick infestation indicating that ticks acquired hosts and were killed or engorged and returned to the kennel. It is hypothesized that engorged ticks located an insufficiently protected skin area on repellent-treated dogs and were able to successfully feed.

Failure to eliminate all premises ticks allows a continued risk of tick-borne disease. Ehrlichiosis is frequently spread by *R. sanguineus* s.l. and this infection can, under some circumstances, transmit relatively quickly into the dog following tick attachment [20]. The very large number of premises tick life stages shown in the present study that can exist in infested kennels indicate that effective tick control is needed for the entire kennel to control the risk of tick-borne disease transmission.

## Conclusions

Fluralaner treatment eliminated off-host *R. sanguineus* s.l. life stages in infested kennels by Day 70 following treatment and was significantly more effective than imidacloprid/flumethrin collar treatment in reducing the premises population of engorged female ticks. Imidacloprid/flumethrin treatment reduced but never eliminated premises tick populations, and these populations increased before the end of the study period.

## Data Availability

All data remain the property of Merck Animal Health, Rahway, NJ, USA. Supplemental data can be made available to selected researchers following receipt of a reasonable request.
